# Effect of Controlling Nb Content and Cooling Rate on the Microstructure, Precipitation Phases, and Mechanical Properties of Rebar

**DOI:** 10.3390/ma17071545

**Published:** 2024-03-28

**Authors:** Bin Shen, Shangjun Gu, Jie Wang, Fulong Wei, Zhiying Li, Zeyun Zeng, Junxiang Zhang, Changrong Li

**Affiliations:** 1 College of Materials and Metallurgy, Guizhou University, Guiyang 550025, China; shenbin775807216@163.com (B.S.); lizhiying2015@163.com (Z.L.); tianyoow@163.com (Z.Z.); yhj1394542756@163.com (J.Z.); 2 Guizhou Province Key Laboratory of Metallurgical and Process Energy Saving, Guiyang 550025, China; 3 Shougang Shuicheng Iron and Steel (Group) Co., Ltd., Liupanshui 553000, China; 13453279792@163.com (S.G.); zzy19950406@163.com (J.W.); nzs18893494794@163.com (F.W.)

**Keywords:** microstructure, texture, precipitation phase, mismatch degree

## Abstract

Seismic anti-seismic rebar, as materials for supporting structures in large buildings, need to have excellent mechanical properties. By increasing the Nb content and controlling the cooling rate, the microstructure and precipitation behavior of the steel are adjusted to develop seismic anti-seismic rebar with excellent mechanical properties. Scanning electron microscopy (SEM), electron backscatter diffraction (EBSD), transmission electron microscopy (TEM), and a universal tensile testing machine were used to characterize the microstructure, precipitation phases, and mechanical properties of the experimental steels. The results show that the ferrite grain size, pearlite lamellae layer (ILS), and small-angle grain boundaries (LAGB) content of the high-Nb steels decreased to 6.39 μm, 0.12 μm, and 48.7%, respectively, as the Nb content was increased from 0.017 to 0.023 wt.% and the cooling rate was increased from 1 to 3 °C·s^−1^. The strength of the {332}<113>_α_ texture is the highest in the high-Nb steels. The precipitated phase is (Nb, Ti, V)C with a diameter of ~50 nm, distributed on ferrite, and the matrix/precipitated phase mismatch is 8.16%, forming a semicommon-lattice interface between the two. The carbon diffusion coefficient model shows that increasing the Nb content can inhibit the diffusion of carbon atoms and reduce the ILS. The yield strength of the high-Nb steel is 556 MPa, and the tensile strength is 764 MPa.

## 1. Introduction

Seismic anti-seismic rebar, also known as high-strength low-alloyed steel (HSLA), have mechanical properties far beyond those of ordinary steels by adding microalloying elements such as Nb, V, Ti, etc. to the steel with a subsequent thermo-mechanical control process (TMCP). The Nb, V, and Ti can combine with the C and N elements to form a fine MC precipitation phase. During the γ→α transformation process, the MC phase can pin down the migration of austenite grain boundaries, inhibit dynamic recrystallization, improve hydrogen embrittlement resistance, etc. [1,2,3,4,5,6]. Therefore, it is essential to study the size, morphology, distribution, orientation relationship (OR), and mismatch relationship of the MC phase.

The shape of the precipitated phase is usually elliptical and square, and the average particle size is about 20–50 nm [7]. Selected area electron diffraction (SAED) results show that the lattice type of the precipitated phase is face-centered cubic [8]. According to the size of precipitates, the OR between precipitates and matrix can be divided into a Kurdjumov–Sachs (K-S) relationship (>15 nm) or Baker–Nutting (B-N) relationship (<15 nm) [9]. Yong et al. [10] provided the formula for the precipitate–temperature–time (PTT) curve, and Ti(C, N) was the first to precipitate, and the rate of precipitation was much greater than that of Nb and V. The results of atomic probe technique (APT) by Webel et al. [11] proved that the Nb–Ti-composite-precipitated phase is a core–shell structure. TiN is the core of the precipitate, and Nb encapsulates TiN to form the shell of the precipitated phase. Liu et al. [12] showed that the Nb-precipitated phase is disk-shaped or near-spherical and distributed inside the grains and in the grain boundaries. The grain boundary precipitation size is larger than the grain interior. Under the same processing conditions, increasing the Nb content can improve the yield of the precipitated phase [13]. According to the new national standard (GB/T 1499.2-2018), the microstructure of seismic rebar can only be ferrite and pearlite [14]. The TMCP process can adjust the type and size of the microstructure of the steel bar and control the distribution and size of the precipitates by controlling the process parameters such as cooling rate, transition temperature, and deformation. Air cooling is the most common cooling method that can inhibit the formation of bainite and increase the content of pearlite [15]. Pan et al. [16] showed that the microstructure of rebar consists of ferrite, pearlite, and bainite when the cooling rate is less than 5 °C·s^−1^, and the microstructure changes to martensite and bainite when the cooling rate is 20 °C·s^−1^. Olasolo et al. [17] showed that when the transformation temperature was 600–650 °C, the content of ferrite and pearlite in the steel was the largest, and the hardness reached the peak. Zhang et al. [18] showed that ferrite and pearlite exhibit different deformation behaviors during the deformation process. At the early stage of strain, ferrite preferentially undergoes plastic deformation, and pearlite deformation is not obvious; at the late stage of strain, the deformation behavior is co-deformation of all microstructures.

The effects of Nb content and cooling rate on the microstructure, precipitation behavior, and mechanical properties of rebar have not been sufficiently investigated, especially the orientation relationship between the precipitated phases and the matrix and the superposition mode of the strengthening mechanism. Therefore, in this paper, two Nb contents and five cooling rates were designed to investigate the variation of ferrite grain size and ILS with Nb content and cooling rate during the continuous cooling process, and the carbon diffusion model was used to explain the effect of Nb content on the pearlite growth behavior. The matrix/precipitate mismatch was calculated. The contribution of four reinforcement increments to yield strength is calculated by using vector superposition, providing a theoretical basis and reference for the development of a new generation of seismic anti-seismic rebar.

## 2. Experimental Method

### 2.1. Experimental Material

The test material was an HRB500E steel bar produced by a factory. Two different Nb-containing steels (0.017 wt.% and 0.023 wt.%), named LN and HN, were designed; 100 t LD converter smelting was used, with argon blowing and stirring of the converter and simultaneous addition of Nb, V, and Ti, and the ingots were melted and poured into 15 kg ingots in an intermediate frequency induction furnace. The ingot was heated to 1200 °C and held for 5 min for homogenization and then cooled down to 1000 °C and 900 °C at a rate of 5 °C·s^−1^ for two passes of hot forging with deformation of 40% and 30%, respectively, followed by air cooling. The chemical composition of the ingots was characterized using a carbon and sulfur analyzer, a hydrogen oxygen nitrogen analyzer, and a direct-reading analyzer, and the chemical composition is shown in Table 1.

### 2.2. Thermal Simulation Process

**Figure 1:**
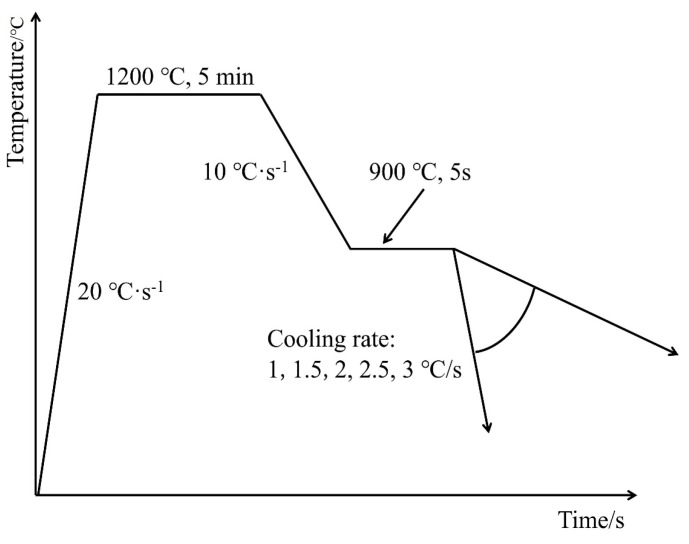
Static thermal simulation process.

The forging sample was processed into a thermal simulation sample of φ8 mm × 15 mm, and the thermal simulation process was carried out on a Gleeble3800 thermal simulation machine. The experimental process is shown in Figure 1.

The specimens were heated on a Gleeble-3800 (Data Sciences International, Albany, NY, USA) thermal simulator at 20 °C·s^−1^ to 1200 °C for 5 min. According to the solid solubility theory, when the holding temperature is 1200 °C, the austenite is homogenized and the microalloying elements can be sufficiently dissolved [13,14,15,16]. Second, it was cooled to 900 °C at 10 °C·s^−1^ for 5 s and then cooled at different cooling rates (1 °C·s^−1^, 1.5 °C·s^−1^, 2 °C·s^−1^, 2.5 °C·s^−1^, and 3 °C·s^−1^) to record the thermal expansion curves.

### 2.3. Characterization Method

The specimens with different cooling rates were etched using a 4% nitric acid alcohol solution, and the microstructure of the experimental steels was characterized using SEM (SUPRA40, Carl Zeiss AG, Oberkochen, Germany). The average ferrite grain size and ILS and the ratio of ferrite to pearlite were counted using the linear intercept method. The samples were machined into discs with a diameter of φ5 mm × 2 mm and polished to a 0.01 mm thickness with 200# metallographic sandpaper. The precast samples were mechanically thinned to 30 μm and then ionically thinned to 0.3 μm. The morphology and composition of the microstructures and precipitates were characterized using TEM (Tecnai G2 F20 S-TWIN, FEI, Hillsboro, OR, USA) equipped with an Energy Dispersive Spectrometer (EDS). Raw high-resolution images (HRTEM) of the precipitates were processed using the HRTEM filter function of the GMS-3 (GATAN, Pleasanton, CA, USA) software, and electron diffraction spots and lattice fringes of the matrix and precipitated phases were obtained using fast Fourier transform (FFT) and inverse fast Fourier transform (IFFT). The electron diffraction spots were calibrated, and the lattice spacing was measured to obtain the corresponding crystallographic indices. EBSD analysis was performed using an EDAX Hikari Plus (EDAX Hikari Plus, EDAX Inc., Warrendale, PA, USA) with the sample rolling direction aligned with the physical *x*-axis of the SEM, and the results were analyzed using OIM to obtain the corresponding rolled surface data. The thermally simulated samples with different cooling rates were processed as plate tensile specimens, and the specimens were stretched at a strain rate of 10^2^∙s^−1^ on an MTS810 universal tensile tester (MTS810, MTS Systems Corporation, Eden Prairie, MN, USA).

## 3. Results and Analysis

### 3.1. Effect of Cooling Rate on Microstructure of Experimental Steel

**Figure 2:**
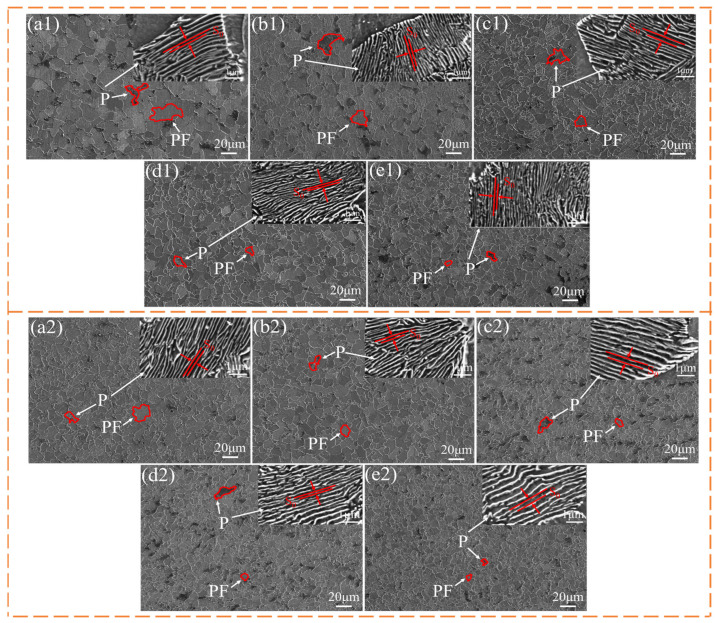
SEM of the microstructure of experimental steel at different cooling rates: (**a1**–**e1**) LN; (**a2**–**e2**) HN.

Figure 2 shows the microstructure of the experimental steels with different cooling rates.

**Figure 3:**
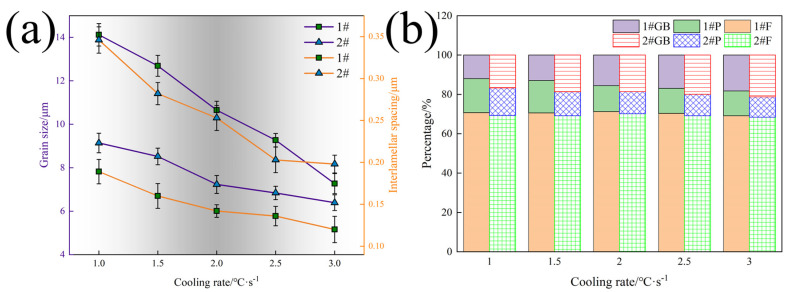
Microstructure statistics of experimental steels: (**a**) grain size and ILS; (**b**) microstructure statistics.

Figure 2 presents that the microstructure of steel at different cooling speeds consists of polygonal ferrite (PF) and pearlite (P). The distribution of polygonal ferrite and pearlite is relatively uniform, and the proportion of ferrite grains is relatively large. With the increase in cooling rate, the grain size of the experimental steel is significantly decreased. Figure 3 shows the grain size, pearlite interlamellar spacing (ILS), and microstructure statistics of the experimental steel.

**Figure 4:**
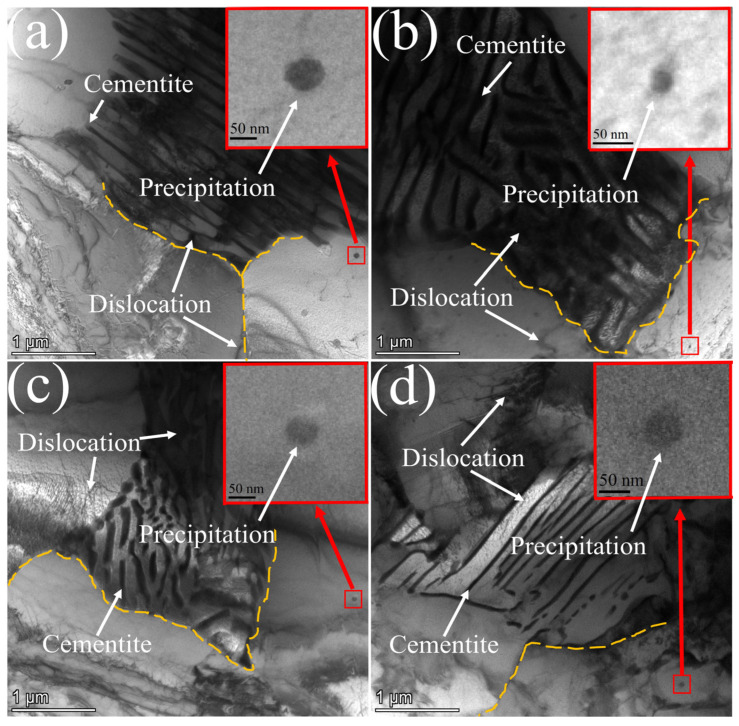
TEM of experimental steels with different cooling rates: (**a**) LN 1 °C·s^−1^; (**b**) LN 3 °C·s^−1^; (**c**) HN 1 °C·s^−1^; (**d**) HN 3 °C·s^−1^ (yellow dashed line is the interface).

Figure 3 presents that with the increase in cooling rate from 1 to 3 °C·s^−1^, the ferrite grain and ILS of the two experimental steels decrease. The ferrite grain size of LN steel decreased by 6.85 μm, the ILS decreased by 0.069 μm, and the proportion of GB increased by 6.27%. The ferrite grain size of HN steel decreased by 2.75 μm, the ILS decreased by 0.148 μm, and the proportion of GB increased by 4.49%. The initial ferrite grain size of HN steel is smaller than that of LN steel, which may be because the Nb content in HN steel is higher than that in LN steel. Nb mainly exists in the austenite GB in the steel as Nb (C, N). These fine precipitates and a small amount of solid-solution Nb atoms can pin the GB migration, prevent the austenite grain growth, and increase the GB content. Figure 4 is the TEM of the experimental steel.

**Figure 5:**
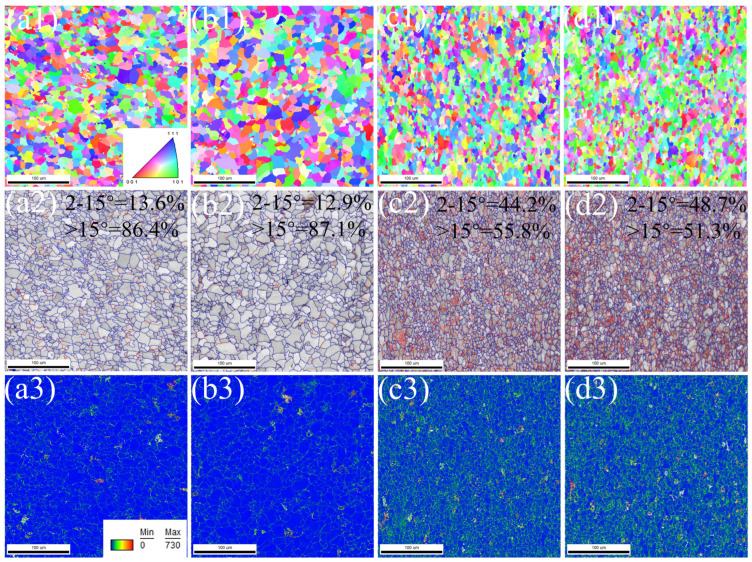
IPF, GB, and GND of experimental steels: (**a1**–**a3**) LN 1 °C·s^−1^; (**b1**–**b3**) LN 3 °C·s^−1^; (**c1**–**c3**) HN 1 °C·s^−1^; (**d1**–**d3**) HN 3 °C·s^−1^.

Figure 4 shows that a large number of dislocations exist between the ferrite and pearlite lamellae. The dislocations are most densely accumulated between the pearlite lamellae. The smaller the spacing of the pearlite lamellae, the more difficult it is for dislocations to slip in between them. At the same time, precipitated phases with a size of ~50 nm are distributed on the ferrite. The fine precipitated phases can pin the moving dislocations and improve the strength of the experimental steel. Figure 5 shows the different cooling rates, inverse pole figure (IPF), GB, and geometrically necessary dislocation density (GND) of the experimental steel.

**Figure 6:**
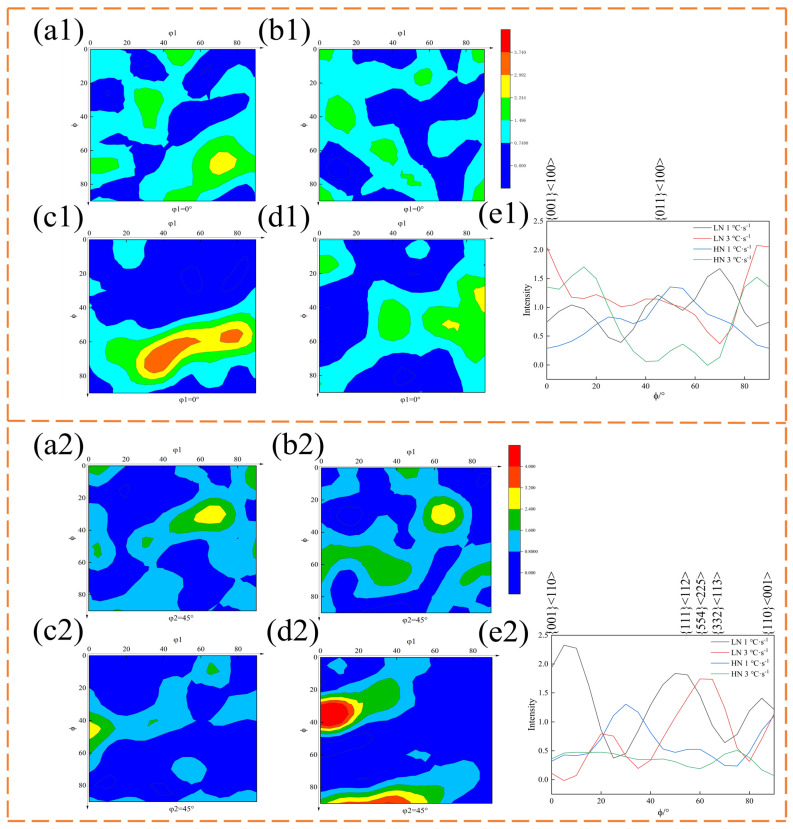
(**a1**–**d1**) ODF at φ_2_ = 0° section. (**a1**) LN 1 °C·s^−1^; (**b1**) LN 3 °C·s^−1^; (**c1**) HN 1 °C·s^−1^; (**d1**) HN 3 °C·s^−1^; (**e1**) η-fiber; (**a2**–**d2**) ODF at φ_2_ = 45° section. (**a2**) LN 1 °C·s^−1^; (**b2**) LN 3 °C·s^−1^; (**c2**) HN 1 °C·s^−1^; (**d2**) HN 3 °C·s^−1^; (**e2**) e-fiber.

From the IPF, it can be visualized that the grain size decreases significantly with the increase in the added Nb content in the steel. The solid solubility of Nb in the steel is extremely small, while Nb is a strong carbide nitride element, which can form precipitation phases in the steel. In the γ→α transformation process, the fine Nb (C, N) distributed in the GB can pin the migration of the GB, preventing the austenite grain growth and increase the grain boundary content. Figure 5(a2–d2) show that with the increase in cooling rate and Nb content, the content of high-angle GB (blue line) decreases from 86.4% to 51.3%, and the content of low-angle GB (red line) increases from 13.6% to 48.7%. Figure 5(a3–d3) show that the increase in Nb content can inhibit the recrystallization behavior of deformed grains. Due to the small orientation difference between adjacent grains (2–15°), local slip occurs when dislocation slips through grains, which is prone to increase local dislocation density. As a result, GND in HN steel is much larger than that in LN steel. The results show that different types of texture can affect the mechanical properties of the experimental steel. Figure 6 shows the orientation distribution function (ODF) and the η-fiber and ε-fiber strengths of the texture for the experimental steels φ_2_ = 0° and φ_2_ = 45°.

The ODF in Figure 6 indicates the presence of texture in the experimental steel. Figure 6(e1) shows that the intensity of {001}<100> texture and {011}<100> texture on η-fiber increases with the increase in cooling rate. Among them, the cooling rate has the greatest influence on the {001}<100> texture. Figure 6(e2) demonstrates that for the ε-fiber, the intensity of the {001}<110> texture in LN steel decreases to a minimum with the increase in cooling rate. When the Nb content in the steel increases, the {001}<110> texture intensity of HN steel increases slightly. The content of {111}<112> texture decreases with the increase in cooling rate and Nb content. Nb content has a great influence on the Goss-{110}<001> texture, and the difference of texture intensity between the two cooling rates in HN steel is greater than that in LN steel. Studies have shown that the static recrystallization of ferrite transforms {111}<110> into {111}<112>. The Brass-{110}<112>_γ_ is transformed into {332}<113>_α_. The transformation of these orientations follows the following path: {332}<113>_α_→{554}<225>_α_→{111}<112>_α_. {332}<113> is an ideal texture, which can significantly improve the plasticity and yield strength of the experimental steel [19].

### 3.2. Effect of Cooling Rate on the Second Phase Precipitation of Experimental Steel

**Figure 7:**
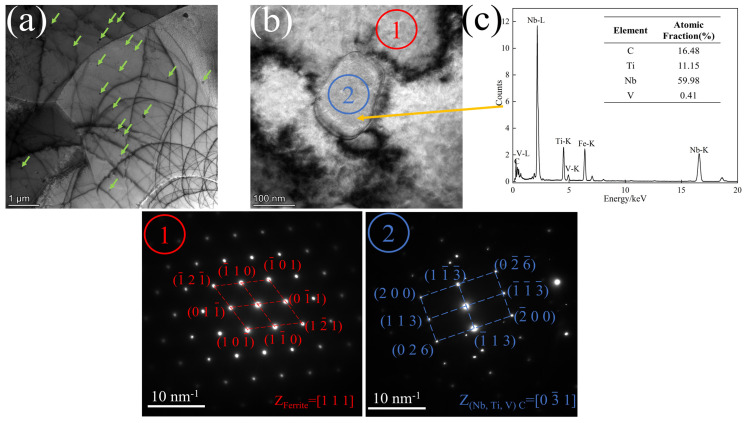
(**a**) Brightfield phase of precipitate distribution; (**b**) TEM of precipitates; (**c**) EDS of precipitates (green arrows point to the precipitates, and (**①**) and (**②**) are the SAEDs at the corresponding positions in (**b**), respectively).

The precipitated phases formed by the microalloying elements Nb, V, and Ti are the strengthening phases in the steel and are characterized using TEM at different locations and with different compositions. The results are presented in Figure 7.

**Figure 8:**
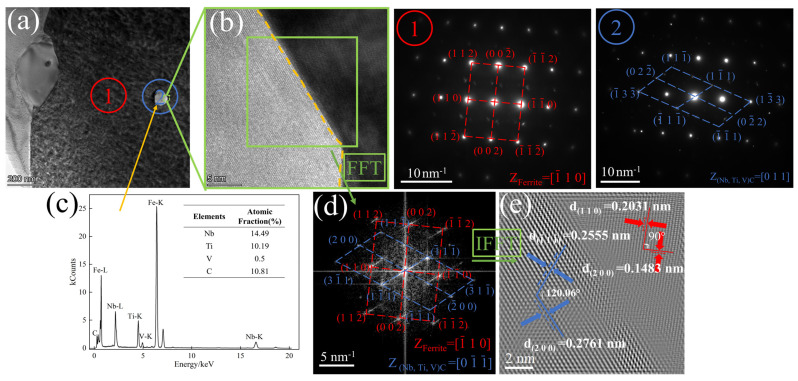
(**a**) TEM of the precipitates; (**b**) HRTEM of the precipitates interface; (**c**) EDS of the precipitates; (**d**) FFT of the precipitates interface; (**e**) IFFT of the precipitates interface ((**①**) and (**②**) are the SAEDs of the corresponding positions in (**a**), and the yellow dash line is the interface, respectively).

Figure 7a illustrates the distribution of a significant number of precipitated phases on the ferrite matrix, with some of them forming on dislocations. Based on the nucleation theory, dislocations can provide energy for the nucleation of precipitated phases. On the other hand, the formed precipitated phases can pin the movement of dislocations and improve the strength of steel. Figure 7b,c demonstrate that the precipitated phase exhibits a square shape with an approximate size of 50 nm. The main components of the precipitated phase are carbides of Nb, V, and Ti, with Nb content being the highest and V content being the lowest. The SAED in the corresponding region of Figure 6(c1,c2) shows that the matrix of the precipitated phase is ferrite with a crystallographic band axis [111]. The precipitated phase is (Nb, V, Ti)C with crystallographic band axis $\left[0\bar{3}1\right]$. Wang et al. [20] showed that there is a certain OR between the precipitated phase and the matrix. The HRTEM of the matrix/precipitate interface and the FFT and IFFT of the corresponding region are shown in Figure 8.

In Figure 8, the matrix is α-Fe, and the crystal band axis is $\left[\bar{1}10\right]$. FFT and IFFT reveal that the crystal planes are (100) and (200), and the crystal plane spacing is 0.2031 nm and 0.1483 nm, respectively. The crystal plane angle is 90°. EDS results indicate that the precipitated phase is (Nb, V, Ti)C, the crystal band axis of the precipitate is $\left[0\bar{1}\bar{1}\right]$, the crystal plane is $\left(\bar{1}1\bar{1}\right)$ and (200), and the crystal plane spacing is 0.2555 nm and 0.2761 nm, respectively. The crystal plane angle is 120.06°. By employing Bramfitt’s two-dimensional mismatch theory [21], the mismatch between the different crystal planes of the precipitate and the matrix was calculated, and the results are presented in Table 2.

Because the mismatch is less than 12%, it can be used as a heterogeneous nucleation interface. Therefore, the (200) surface of α-Fe is combined with the (200) surface of the precipitate.

### 3.3. Effect of Cooling Rate on Mechanical Properties of Experimental Steel

**Figure 9:**
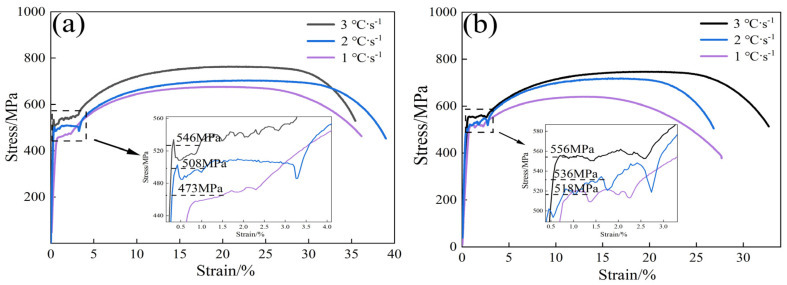
Tensile curves of two Nb-containing steels at different cooling rates: (**a**) LN; (**b**) HN.

Cooling rates of 1, 2, and 3 °C·s^−1^ experimental steels were selected for tensile tests, and the engineering stress–strain curves of the experimental steels are shown in Figure 9. Table 3 shows the stress–strain data statistics.

As depicted in Figure 9 and Table 3, both the tensile and yield strengths of the two Nb-containing steels increase with the rise in cooling rate and Nb content. When the cooling rate is increased to 3 °C·s^−1^, the yield strength of LN steel increases to 546 MPa and the tensile strength to 747 MPa, while the yield strength of HN steel increases to 556 MPa and the tensile strength to 764 MPa. Studies have shown that increasing the cooling rate can reduce the grain size, retain more grain boundaries, and improve the effect of GB strengthening [7].

## 4. Discussion

### 4.1. Continuous Cooling Transition Curve and Diffusion Coefficient Curve

**Figure 10:**
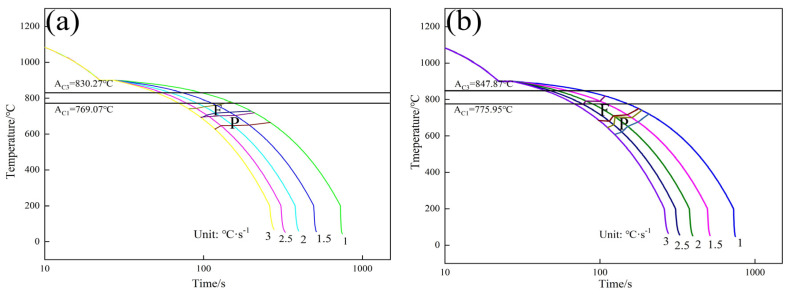
CCT curves of experimental steels: (**a**) CCT curve of LN steel; (**b**) CCT curve of HN steel.

The continuous transformation curve of the experimental steel is shown in Figure 10.

Figure 10 shows that with the increase in Nb content, the temperatures of A_C1_ and A_C3_ increase slightly, and the starting time of pearlite transformation of the experimental steel is delayed. This is because the pearlite is a diffusion phase transition, and the Nb atom drags the diffusion of the carbon atom. Lee et al. [22] considered the hindering effect of Nb on the migration of carbon atoms and modified the equation for the carbon diffusion coefficient (Equation (1)):

(1) $$D_{0}{=D}_{C}^{0}{\exp(5000X}_{M}(\frac{2750}{T}-1.85))$$

where X_M_ is the molar fraction of Nb, V, and Ti, and $D_{C}^{0}$ denotes the diffusion coefficient of carbon atoms without added microalloying elements. 

**Figure 11:**
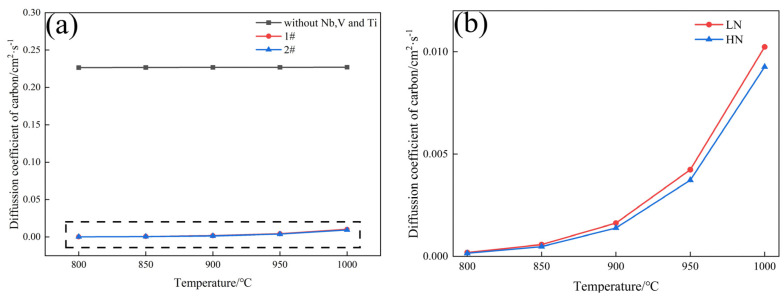
Diffusion coefficient curve of experimental steel ((**b**) is the enlarged picture of the selected area of the black dotted line in (**a**)).

Figure 11 shows that the addition of microalloying elements can significantly reduce the diffusion coefficient of carbon atoms, and with the increase in Nb content, the diffusion coefficient of carbon atoms of HN steel is lower than that of LN steel. Cementite is the main reinforcing phase in pearlite, and increasing the Nb content can reduce the growth rate of cementite and decrease the thickness of cementite. Studies have shown that when the thickness of the cementite is less than 200 nm, the cementite will bend and rotate during stretching rather than break [23].

### 4.2. Strengthening Mechanisms

The four common strengthening mechanisms are solid solution strengthening (σ_SS_), fine-grain strengthening (σ_GB_), precipitation strengthening (σ_PR_), and dislocation strengthening (σ_Dis_). There is a relationship between them and the yield strength, as shown in Equation (2):

(2) $$\sigma_{y}{=\sigma }_{0}{+\sigma }_{\mathrm{SS}}{+\sigma }_{\mathrm{GB}}{+\sigma }_{\mathrm{PR}\;}{+\sigma }_{\mathrm{Dis}}$$

Yong et al. [7] pointed out that grain boundaries can be considered as dislocations with a total length of L, and precipitates can also increase dislocation density, so dislocation strengthening and fine-grain strengthening use root-mean-square superposition.

(3) $$\sigma_{y}{=\sigma }_{0\;}{+\sigma }_{\mathrm{SS}}{+\sigma }_{\mathrm{PR}}+\sqrt{\sigma_{\mathrm{GB}}^{2}{+\sigma }_{\mathrm{Dis}}^{2}}$$

where σ_0_ is the friction stress, which has a value of 53.9 MPa [7]. The calculation formulas of the four strengthening effects are as follows [10,24,25]:

(4) $$\sigma_{\mathrm{SS}}=37[\mathrm{Mn}]+83[\mathrm{Si}]+470[P]30[\mathrm{Cr}]$$

(5) $$\sigma_{\mathrm{GB}}{=\sigma }_{f}V_{f}+\sigma_{p}V_{p}$$

(6) $$\sigma_{\mathrm{PR}}=8{.995\;\times \;10}^{3}\frac{f_{v}^{1/2}}{d_{\mathrm{PR}}}\ln(2{.417d}_{\mathrm{PR}})$$

(7) $$\sigma_{\mathrm{Dis}\;}{=\alpha \mathrm{MGb}\rho }^{1/2}$$

where [Mn], [Si], [P], and [Cr] are the mass fractions (wt.%) of the corresponding elements in the steel. σ_f_ and σ_p_ are the contributions of ferrite and pearlite to strength, and V_f_ and V_p_ are the percentages of ferrite and pearlite in the experimental steel [24]. 

d_PR_ is the average precipitate size (~50 nm), and f is the volume fraction of the Nb/Ti precipitates in the ferrite (%). α is the constant 0.24 [26], M is the deformation-induced orientation factor of about 2 [24], G is the shear modulus (81,600 MPa for steel) [26], b is the Burgers vector (0.248 nm) [27], and ρ is the dislocation density in the steel.

The strength of ferrite and pearlite can be calculated by Equation (8) [10]:

(8) $$\sigma_{i(i=f,\;p)}{=k}_{\mathrm{HP}}\cdot d^{1/2}$$

where k_HP_ is the proportionality constant and takes the value of 17.4 MPa·mm^0.5^ [28]; d is the average ferrite grain size (nm) and ILS (nm). 

The volume fraction of (Nb, V, Ti)C in the ferrite can be calculated with Equation (9) [29]:

(9) $$f_{v}=\left(\sum M_{i}-\sum \left[M_{i}\right]+C\;-\left[C\right]\right)\frac{\rho_{\mathrm{Fe}}}{{100\rho }_{\mathrm{MC}}}$$

where M_i_ (M_i_ = Nb, Ti) is the content of microalloying elements in steel (wt.%); [M_i_] is the solid solution amount of microalloying elements in ferrite; C is the content of element C in steel (wt.%); [C] is the solid solution amount of C in ferrite; ρ_Fe_ and ρ_MC_ are the densities of Fe and MC (M = Nb, V, Ti), that is, ρ_Fe_ = 7.8 g/cm^2^ and ρ_(Nb,V,Ti)C_ = 6.385 g/cm^2^.

**Figure 12:**
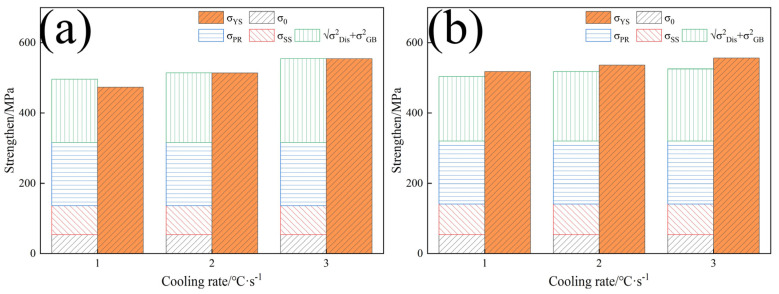
The comparison between the increment of strengthening mechanism and yield strength: (**a**) LN; (**b**) HN.

Figure 12 shows the strengthening mechanism versus yield strength.

Figure 12 demonstrates that the theoretical calculation results align well with the actual yield strength, suggesting that Equation (3) is more consistent with the experimental findings. Similar results have been reported by other researchers [11].

## 5. Conclusions

The microstructure, texture content, orientation relationship between α-Fe and precipitated phase, and mechanical properties of the experimental steel were characterized by SEM, EBSD, TEM, and universal tensile testing machine. The microstructure ratio and ILS were counted. The matrix/precipitate mismatch and carbon diffusion coefficient were calculated. The strengthening mechanism of the experimental steel was discussed, and the conclusions are as follows:

1. As the Nb content increased from 0.017 to 0.023 wt.%, the ferrite grain size of the experimental steels decreased to 9.14, 8.52, 7.23, 6.84, and 6.39 μm for the five cooling rates (1, 1.5, 2, 2.5, and 3 °C·s^−1^, respectively), and the ILS decreased to 0.189, 0.160, 0.142, 0.136, and 0.12 μm, respectively. The {332}<113>_α_ texture strength of HN steel increased, and the carbon diffusion coefficient decreased.
2. A large number of nanoscale precipitated phases are distributed on the grain boundaries and dislocations of ferrite, which provide a non-uniform nucleation core for the precipitates. The precipitates are mainly (Nb, Ti, V)C carbides with a diameter of ~50 nm. The mismatch between the matrix and the precipitated phase is 8.16%. Both matched crystal faces are (200). A semicommon-lattice interface is formed between the two.

With the increase in Nb content from 0.017 to 0.023 wt.%, the cooling rate increased from 1 °C·s^−1^ to 3 °C·s^−1^. The yield strength and tensile strength of LN steel reached 546 MPa and 747 MPa, respectively. The yield strength and tensile strength of HN steel reached 556 MPa and 764 MPa, respectively. The results of the strengthening mechanism show that the vector sum of dislocation strengthening and grain boundary strengthening is closer to the experimental results.

## Figures and Tables

**Table 1:** Chemical composition of the steels (wt.%).

Steel	C	Si	Mn	P	S	Nb	V	Ti	N
LN	0.16	0.30	1.31	0.019	0.013	0.017	0.085	0.007	0.009
HN	0.17	0.34	1.35	0.019	0.012	0.023	0.085	0.007	0.009

**Table 2:** Mismatch degree of different crystal planes and substrates of precipitates.

d_matrix_/nm	d_precipation_/nm	θ/°	d_precipation_cosθ	δ/%
0.1483	0.2761	22.67	0.2547	23.9
0.2013	0.2555	53.47	0.1520	8.16

**Table 3:** Mechanical properties of two Nb-containing steels at different cooling rates.

Cooling Rate/°C·s^−1^	R_eL_/MPa	R_m_/MPa	R_m_/R_eL_	A/%
LN-1.0 °C·s^−1^	473 ± 9	641 ± 7	1.24	27.66 ± 1.6
LN-2.0 °C·s^−1^	508 ± 6	719 ± 6	1.34	26.85 ± 1.8
LN-3.0 °C·s^−1^	546 ± 6	747 ± 6	1.34	32.73 ± 1.6
HN-1.0 °C·s^−1^	518 ± 6	675 ± 7	1.42	36.15 ± 1.7
HN-2.0 °C·s^−1^	536 ± 7	704 ± 5	1.39	38.99 ± 1.7
HN-3.0 °C·s^−1^	556 ± 6	764 ± 6	1.40	32.44 ± 1.6

## Data Availability

Data are contained within the article.
